# Nutrient, non-nutrient, antioxidant activity, and Fourier Transform Infrared analysis of Kenyan indigenous edible leafy vegetables from *Launaea cornuta* (Hochst Ex Oliv and Hiern)

**DOI:** 10.4314/ahs.v23i4.56

**Published:** 2023-12

**Authors:** Jared Misonge Onyancha, Gervason Apiri Moriasi, Elias Nelson Mandela

**Affiliations:** 1 School of Pharmacy, Department of Pharmacognosy, Mount Kenya University, PO Box 01000-342, Thika, Kenya; 2 School of Medicine, Department of Medical Biochemistry, Mount Kenya University, PO Box 01000-342, Thika, Kenya; 3 School of Pure and Applied Sciences, Department of Biological Sciences, Mount Kenya University, PO Box 01000-342, Thika, Kenya

**Keywords:** Kenya, mineral elements, phytochemicals, proximate analysis, traditional medicine

## Abstract

**Background:**

*Launaea cornuta* is a vegetable with therapeutic advantage for human diseases.

**Objective:**

Evaluate nutritive and non-nutritive components, antioxidant activity, and Fourier transform infrared spectroscopy profile of L. cornuta leaves.

**Methods:**

Proximate, nutri, non-nutrient, percentage phenolic, flavonoid, alkaloid, and saponin contents were investigated using standard procedures. Total phenolic and flavonoids of the extracts were determined spectroscopically. Antioxidant activity and functional groups in the extracts were characterised by 2.2- diphenyl-2-picryl-hydrazyl radical and FTIR spectroscopy, respectively.

**Results:**

Carbohydrates were the most abundant (57.61±0.61 %), and crude lipids were the least abundant (4.26±0.20 %) in *L. cornuta*. Essential amino acids were present in varying concentrations, and histidine was the most abundant (251.20±2.00 mg/100 g dw). Calcium was the most abundant mineral element (820.49±1.05 µg/g dw). High concentrations of phenols (13.07±0.60 %) and low amounts of saponins (2.19±0.10 %) were recorded. Methanolic and aqueous leaf extracts revealed total phenols of 83.10±4.32 and 57.77 ±1.65 mgGAE/g dw, respectively, while total flavonoids were 8.00±0.01 and 7.99±0.03 mgCE/g of dry weight, respectively. Aqueous extract had significant DPPH radical scavenging efficacy (IC50 =72.96± 0.32 µg/ml) compared to 681.57± 2.21 jg/ml for methanol extract.

**Conclusions:**

*L. cornuta* contain phytochemicals with health benefits for averting oxidative stress related diseases.

## Introduction

Vegetables are edible parts of plants and usually comprise leaves, roots, fruits, or seeds. Over 10,000 wild plant species are used as vegetables and staple food, with low calories and high nutrients like dietary fibres, vitamins, minerals, and non-nutritive phytochemicals, including phenolic compounds, bioactive peptides, carotenoids, phytosterols, and organosulfur compounds^1^. The nutritive and non-nutritive (antinutritive) vegetable bioactive compounds confer significant health benefits such as antioxidant, antibacterial, and enzyme stimulators, enhancing health, modulating immunity, and mitigating various diseases, including chronic and devastating syndromes^2^. Vegetables have played a central role in food and medicine since prehistoric times and are still crucial in reducing food insecurity and maintaining human health in economically developing countries 3. In Africa, especially Kenya, indigenous leafy vegetables form the main part of daily diets and are preferred due to their nutritive and medicinal value^4^.

**Plate 1:**
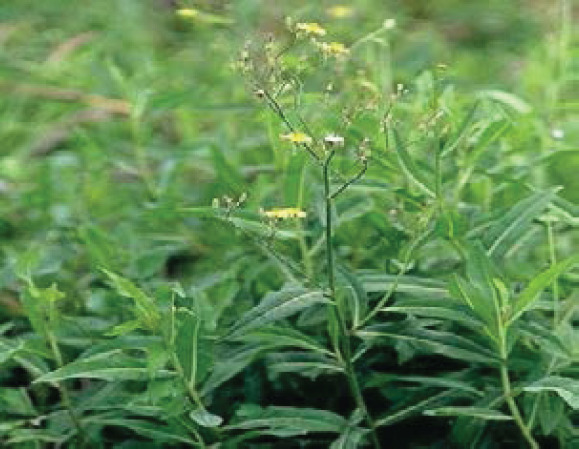
A photograph showing aerial floral parts of *L. cornuta* (captured in Situ by the authors)

**Figure 1:**
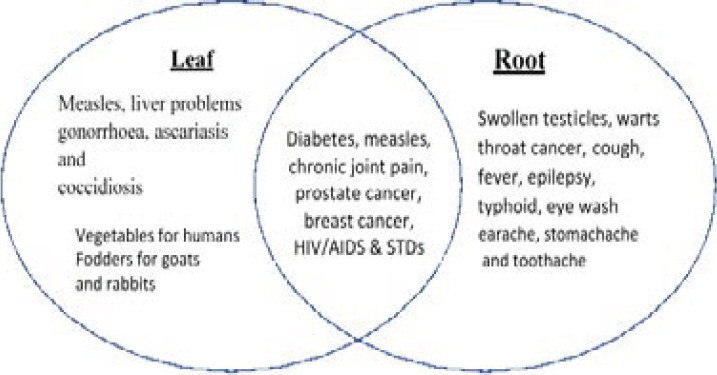
Summary of the ethnomedicinal uses of *L. cornuta* leaves and roots

*Launaea cornuta* (Hochst. Ex Oliv. and Hiern.) (Plate 1) is a wild and neglected leafy vegetable commonly known as bitter lettuce^5^. It is an erect perennial herb with milky juice, hollow stems up to 1.5 m high, and creeping rhizomes. It is indigenous to Kenya, Uganda, Malawi, Tanzania, Mozambique, and Zimbabwe. It is resistant to drought and is used as a source of traditional vegetables by the Kamba, Kikuyu, Luo, Giriama, and Taita communities in Kenya, some Coastal tribes in Somalia, and the Sambaa people of Tanzania^6^,^7^. The leaves of *L. cornuta* are considered a rich source of nutrients such as proteins, fat, carbohydrates, calcium, phosphorus, iron, and ascorbic acid 6. *L. cornuta* is used ethnomedicinally as an antidiabetic 8, anticancer 9, insecticidal 10, antimalarial, antibacterial 11, and anthelmintic^12^ remedy among various communities. It has also been indicated for treating gonorrhoea, syphilis, sore throats, coughs, typhus, nasal-pharyngeal infections, fever 13, throat cancer 14, measles and swollen testicles 15, spleen pain, hookworm infestation, and chronic joint pains^16^ in traditional medicine. The ethnomedicinal uses of the *L. cornuta* plant are summarised in Figure 1. Recently, Akimat et al.^17^ demonstrated the *in vitro, ex vivo*, and *in vivo* anti-inflammatory and analgesic efficacy of the aqueous root extract of *L. cornuta*, depicting its medicinal value.

Although *L. cornuta* leaves are extensively utilised as food and medicine in traditional medicine, there is no sufficient empirical data to validate their nutritional and pharmacological value. Therefore, this study evaluated the nutrient and antinutrient composition, antioxidant activity, and FTIR profile of *L. cornuta* leaves. The current study's findings provide scientific data to support some of the ethnomedicinal claims of *L. cornuta* and lay a foundation for further investigations, which may lead to the isolation and characterisation of bioactive amalgams for drug development.

## Materials and methods

### Plant materials and sample preparation

Fresh leaves of mature *L. cornuta* were collected from Ngoliba in Kiambu County, in August 2019, with the help of a locally renowned herbalist. The herbarium sample was prepared according to standard procedures and was authenticated at the Department of Botany of the East Africa Herbarium, Nairobi, Kenya. Duplicate voucher specimens (JMO-1-2014) were deposited at Mount Kenya University Herbarium at the School of Pharmacy for future reference. The leaves of *L. cornuta* were shade dried in an adequately ventilated drying room at the pharmacognosy laboratory at Mount Kenya University for one week. Occasional grabbling was done to facilitate aeration and thwart moisture build-up. The dried material was then cut into smaller pieces and ground into a coarse powder using an electric hammer plant mill. The ground powder was stored in a closed glass bottle at room temperature awaiting analysis and extraction.

### Extraction procedures

Aqueous and methanolic extracts were prepared according to the hot and cold maceration procedures described by Houghton and Raman 18 and modified by Onyancha *et al.* 19. In brief, approximately 20 g of the powder was transferred into a boiling flask, after which 100 ml distilled water was added, and the mixture heated for 5 minutes in a water bath set at 60 °C. The concoction was cooled at room temperature, after which it was filtered and lyophilised in a freeze drier (Thermo Fisher Scientific) to obtain the aqueous extract. About 100 g of the leaf powder was macerated in analytical-grade methanol (Scharlau) in a 250 conical flask for the methanolic extract. The mixture was shaken, and the flask was covered with aluminium foil paper and allowed to interact for 48 hours with constant stirring using a magnetic stirrer. After that, the extract was filtered and concentrated in *vacuo* at 40 °C and then dried thoroughly in the oven (LabTech) set at 35 °C. The extract was weighed and packaged in labelled, brown-coloured sample bottles, which were tightly closed and kept in a refrigerator at 4 °C awaiting experimentation.

### Proximate analysis

Proximate analysis of carbohydrate content, moisture content, ash content, crude fibre, crude protein, and crude lipid was performed according to the standard analytical procedures described by the Association of Official Analytical Chemists (AOAC) 20 as adopted by Nielsen^21^,^22^ and Udofia *et al.* 21,22

### Determination of the concentration of essential amino acids

Amino acid concentrations in *L. cornuta* leaf powder were determined according to the methods described by Okoronkwo *et al.* 22,23 with slight modifications. Five grams of the leaf powder was weighed and macerated in 50 ml of 95% ethanol in a water bath set at 50 °C for 30 minutes. The mixture was carefully filtered through Whatman filter paper No. 1, and 5 ml aliquots were taken and transferred into clean 50 ml volumetric flasks. Then, 2.5 ml of 0.25% sodium carbonate (LobaChemie) and 5 ml of 1% Ninhyrin (Sigma Aldrich) solution prepared in 95 % ethanol (Scharlau) were added and heated for 5 min in a hot water bath (95 °C) for five minutes, cooled to room temperature and made up to the mark with distilled water. The standard amino acids were prepared similarly to the test samples. The absorbance values were determined using a UV-Vis spectrophotometer (Shimadzu 1601) at wavelengths ranging from 204 nm to 350 nm with distilled water as the blank and used to determine amino acid concentrations in the assay samples using a per cent solution extinction coefficient (e per cent of 10). The percentage (%) concentration of each amino acid $(\frac{Absorbance}{\varepsilon \;\text{percent}})mg/ml$ were then converted to mg/100 g 22.

### Determination of mineral element concentrations

The mineral element composition of *L. cornuta* leaf powder was determined using an atomic absorption spectrophotometer (Thermo-scientific AA 301) using the procedure of Achi *et al.* 24. The dry-ashing sample preparation technique was adopted, whereby 2 g of the plant powder was weighed and ashed by heating in a muffle furnace (Lab-Tech) at 550 °C for five hours. The resultant ash was then left to cool to rom temperature, dissolved in 50 ml of 1% nitric acid, filtered through Whatman No.1 filter paper, and the filtrates made to the 100 ml mark using the same solvent to make the stock solution. The standard solutions of each mineral element were prepared in 1% nitic acid (LobaChemie) solution, and the standard curves of each mineral element were prepared and used to interpolate the values for the plant material. The mineral elements in each sample were analysed in triplicate 22.

### Determination of the quantitative phytochemical composition of L. cornuta leaves

Quantitative determination of alkaloids, saponins, phenols, and flavonoids of *L. cornuta* leave powder was determined using previously described procedures^22^,^25^-^28^.

### Alkaloid

The quantity of alkaloids in the powder of *L. cornuta* leaves was determined according to the methods described by Ezeonu and Ejikeme 26 and Udofia *et al.*^21^ with slight modifications. Briefly, five grams (5 g) of the powder was weighed into a 250 ml beaker, after which 200 ml of 20 % acetic acid (LobaChemie) in ethanol (Scharlau) was added. The mixture was allowed to stand for 4 hours, filtered, and then evaporated to about one-quarter of the original volume in a hot water bath (Lab-Tech) set at 95 °C. Then, concentrated ammonium solution (LobaChemie) was added dropwise to the extract until precipitation was complete. The entire solution was allowed three hours to settle, after which the supernatant was discarded, and the precipitates were washed with 20 ml of 0.1 M of ammonium hydroxide (LobaChemie) and filtered. The precipitate was oven-dried, and the weight of the precipitate was recorded. The percentage alkaloid content was calculated as shown in equation 1 (Eqn. 1).


$$\text{Percentage}\;\text{alkaloid}\;\text{content}=\frac{\text{Weight}\;\text{of}\;\text{the}\;\text{alkaloid}}{\text{Weight}\;\text{of}\;\text{the}\;\text{sample}}\times 100\ldots \ldots \ldots Eqn.\;1.$$

### Saponin

Percentage saponin content was determined using the methods of Kwada and Tella 28 and Ezeonu and Ejikeme^22^,^26^ with slight modifications. In brief, five grams of the powdered material was weighed and dissolved in 50 ml of 20 % ethanol in a 250 ml conical flask. The mixture was heated over a hot water bath (55 °C) for 4 hours with continuous stirring. The mixture was filtered, and the residue was re-extracted with another 50 ml of 20 % ethanol (Scharlau) and further heated over a hot water bath (LobaChemie) at 55 °C for another 4 hours with continuous stirring. The extracts were combined and then reduced to 10 ml over a hot plate (Lab-Tech) at 90 °C. The concentrate was transferred into a 250 ml separating funnel and mixed with 5 ml diethyl ether (Sigma Aldrich). The aqueous layer was recovered, while the ether layer was discarded. Then, 15 ml of n-butanol (Sigma Aldrich) was added to the aqueous layer, and the resultant solution was washed twice with 2.5 ml of 5 % aqueous sodium chloride (LobaChemie). The remaining solutions were evaporated in a water bath and dried to a constant weight in a hot air oven (LabTech). The saponin content was calculated as a percentage using equation 2 (Eqn.2).


$$\text{Percentage}\;\text{saponin}\;\text{content}=\frac{\text{Weight}\;\text{of}\;\text{saponin}}{\text{Weight}\;\text{of}\;\text{sample}}\times 100\ldots \ldots \ldots Eqn.\;2.$$

### Phenolic

Quantitative determination of phenolic content in the powder of *L. cornuta* leaves was determined using the method described by Hussain *et al.*^22^,^29^ with slight modification. Approximately 5 g of leaf powder was weighed into a 250 ml titration flask, and 100 ml n-hexane was added twice and allowed to extract for 4 hours. The mixture was filtered, and the filtrate was discarded for fat-free sample preparation. Diethyl ether (Sigma Aldrich) (100 ml) was added to the residue, heated for 15 minutes, cooled to room temperature, and filtered into a separating funnel. Then, 100 ml of 10 % NaOH (Sigma Aldrich) solution was added and swirled to separate the aqueous and organic layers. The aqueous layer was washed thrice with 25 ml of deionised water and acidified up to pH 4.0 with a 10 % HCl (Sigma Aldrich) solution. After that, 50 ml dichloromethane (Sigma Aldrich) was added to the acidified aqueous layer in a separating flask, mixed, and the organic layer was collected and allowed to dry. The precipitate was weighed (Lab-Tech), and the percentage of phenolic content was calculated using Equation 3 (Eqn. 3).


$$\text{Percentage}\;\text{phenolic}\;\text{content}=\frac{\text{Weight}\;\text{of}\;\text{the}\;\text{phenolics}}{\text{Weight}\;\text{of}\;\text{the}\;\text{sample}}\times 100\ldots \ldots \ldots Eqn.\;3.$$

### Flavonoid

The quantity of flavonoids in *L. cornuta* leaf powder was determined following the method described by Ezeonu and Ejikeme 22,26. Briefly, 5 g of the powder was weighed into a 250 mL volumetric flask, and 100 mL of the 80 % aqueous methanol (Scharlau) was added and shaken for 4 hours in an electric shaker at room temperature. The entire solution was filtered through Whatman filter paper number 1. The filtrate was reextracted using 100 ml of the 80 % aqueous methanol, shaken for 4 hours in an electric shaker then filtered at room temperature. The filtrates were later transferred into pre-weighed crucibles, evaporated to dryness over a hot plate, and re-weighed. The percentage flavonoid content was computed according to equation 4 (Eqn. 4).


$$\text{Percentage}\;\text{flavonoid}\;\text{content}=\frac{\text{Weight}\;\text{flavonoid}}{\text{Weight}\;\text{of}\;\text{sample}}\times 100\ldots \ldots \ldots Eqn.\;4.$$

### Determination of the total phenolic and flavonoid concentration in the aqueous and methanolic leaf extracts of L. cornuta

The total phenolic concentration/content (TPC) in the aqueous and methanolic leaf extracts of *L. cornuta* was determined based on the Folin-Ciocalteu assay method described by Saeed *et al.*^30^ with slight modifications. Briefly, different gallic acid (LobaChemie) concentrations ranged from 150 µg/ml to 4.6875 µg/ml, after which 0.3 ml was drawn from each concentration and mixed with 1.5 ml of Folin-Ciocalteu's phenol reagent (10%) (LobaChemie). Besides, 0.3 ml of each study extract was separately mixed with 1.5 ml Folin-Ciocalteu's phenol reagent (10%) (LobaChemie). After that, 1.5 ml of 7.5% Na_2_CO_3_ (LobaChemie) solution prepared in deionised water was added, and the setups were incubated in a dark room for 2 minutes. Then, the absorbance values of gallic acid and the test extracts were read at 760 nm using a UV-Vis spectrophotometer. The experiment was performed in triplicate. A calibration curve of absorbance versus concentration was drawn using the gallic acid (Loba Chemie) absorbance values and concentrations to interpolate the plant extracts' concentrations. Total phenolic content was calculated using the regression equation for the triplicate experiment was Y = 0.009210*x* + 0.09029, *R*^2^ = 0.9799, where y was the absorbance at 760 nm and x was the concentration of Gallic acid (µg/mL). After that, the TPC of the studied extracts was expressed as milligrams of gallic acid equivalents (GAE) per g of dried sample.

The total flavonoid concentration/content (TFC) of *L. cornuta* leaf extracts was determined using the aluminium nitrate colourimetric method described by Cosmulescu *et al.* 31 with some modifications. In brief, 0.125 ml of 1 % *L. cornuta* leaf extracts in methanol and different concentrations of Catechin (Sigma Aldrich) (20 µg/ml to 0.625 µg/ml) were prepared in triplicate. Then, 0.075 ml of 5 % sodium nitrate (NaNO_3_) (LobaChemie) was added to the test tubes containing the sample and those with different concentrations of the standard (Catechin) (Sigma Aldrich). After that, the mixtures were incubated for six minutes at room temperature and mixed with 0.15 ml of 10% Aluminium nitrate (AlNO_3_) (Loba Chemie) and 0.75 ml of 4% sodium hydroxide. The mixtures were made up to 2.5 ml with distilled water, and their respective absorbances were measured at 510 nm using a UV-Vis spectrophotometer (Shimadzu 1601). The blank solution contained all the reagents except the study extracts and Catechin; instead, an equal volume of methanol was added. The TFC of the study extracts were interpolated from the Catechin standard calibration curve (Y = 0.0208*x* + 0.0149, *R*^2^ = 0.952), where, Y is the absorbance at 510 nm, and x is the concentration of Catechin (µgCE/mL). The experiment was performed in triplicate, and the TFC of the extracts was expressed as mg Catechin equivalent per gram (mgCE/g) of dried plant material.

### Determination of in vitro antioxidant activity of the aqueous and methanolic leaf extracts of L. cornuta

The antioxidant activity of *L. cornuta* leaf extracts was determined using the 2,2-diphenyl-2-picrylhydrazyl (DPPH) free radical scavenging assay method described by Moriasi *et al.*,^32^ with slight modifications. Test concentrations (1000 µg/ml, 100 µg/ml, 10 µg/ml, 1 µg/ml, 0.1 µg/ml, and 0.01 µg/ml) of *L. cornuta* leaf extracts and standard (L- ascorbic acid) were separately prepared in methanol (Scharlau). DPPH (Sigma Aldrich) (0.3 mM) was prepared in methanol, and 2.4 ml was added to 1.6 ml of the extract and the standard (L-ascorbic acid) (Sigma Aldrich) concentrations. The control for the current study consisted of a mixture of 2.4 ml of 0.3 mM DPPH solution in methanol and 1.6 ml of methanol. The test samples and the standard were prepared in triplicate and kept in the darkroom for 15 minutes, after which the absorbances were measured at a wavelength of 517 nm against methanol as the blank using a UV-Vis spectrophotometer (Shimadzu 1601). The percentage radical scavenging activity (% RSA) of each extract and standard was calculated using the formula shown in equation 5 (Eqn. 5).


$$\%\text{RSA}=\frac{\text{Abs.Control}-\text{Abs.Test}}{\text{Abs.Control}}\times 100\ldots \ldots \ldots \ldots \ldots \ldots Eqn.\;5.$$

Where %RSA= Percentage radical scavenging activity; Abs= absorbance; Test=Extract or standard (L-ascorbic acid).

Moreover, the antioxidant efficacy was expressed as a value of the concentration of *L. cornuta* leaf extracts that could scavenge 50 % of the DPPH radicals (IC_50_). IC_50_ values were calculated from the curve of %RSA versus concentrations of the respective extracts and standard. The antioxidant activity levels (efficacy) were classified based on the IC_50_ values as very strong (IC_50_ < 50 µg/ml), strong (50 µg/ml <IC_50_> 100 µg/ml), medium (100 µg/ml <IC_50_> 150 µg/ml), weak (1C_50_ µg/ml <IC_50_> 200 µg/ml) and very weak (IC_50_ > 200 µg/ml) 33.

### Analysis of L. cornuta leaf powder by Fourier transform infrared (FTIR) spectroscopy

Fourier Transform Infrared spectroscopy was performed by adopting the method described by Wangia et al., 2018 34. Briefly, 10 mg of the powdered plant material was mixed with 100 mg of spectroscopic-grade potassium bromide (KBr) (Sigma Aldrich) and ground using a mortar and pestle. The resultant fine powder mixture was compressed using a hand press (5 × 10^6^ Pa) in an evacuated die to produce a clear transparent 1 mm thick disc with a diameter of 13 mm. The disc was placed in a sample handler of the FTIR equipment (Shimadzu-A219651) and scanned twenty times. The produced spectra were recorded and interpreted according to standard guidelines 34.

### Data management and statistical analysis

This study collected quantitative data, entered it into a spreadsheet and analysed using GraphPad Prism software version 8.1. The FTIR spectra were interpreted according to a previously described procedure 34.

## Results and discussion

### Proximate composition of the L. cornuta leaves

The quantitative analysis of L. cornuta leaves revealed macromolecule compositions of carbohydrate, crude fibre, crude protein, and crude lipid. Ash values and moisture content of L. cornuta were also recorded (Table 1).

**Table 1:** Proximate composition of the *L. cornuta* leaves

Proximate parameter	Concentration (%)
Carbohydrates	57.61 ± 0.70
Ash content	14.98 ± 0.30
Moisture content	8.48 ± 0.10
Crude protein	7.97 ± 0.20
Crude fibres	6.70 ± 0.20
Crude lipids	4.26 ± 0.20

The values are presented as *x̅* ± *SEM* of triplicate analyses.

To our knowledge, moisture, ash, and crude fibres are reported for the first time in the current study. However, carbohydrates (4.5 g), proteins (3.9 g), and fat (0.9 g) have been reported previously in young leaves of *L. cornuta* 35. The differences in the reported values of carbohydrates, proteins, and fats could be attributed to several factors, such as time of collection, plant age and climate. The nutritive chemicals confer health benefits to humans and animals. The macronutrients in food sources are an essential package of many different chemicals, including fats, fibre, and sodium. The crude fibres, or roughage, are the indigestible part of food plants, crucial for gut health and reducing the risk of chronic health conditions 36.

### Composition of essential amino acids in L. cornuta leaf

The current study revealed varying quantities of essential amino acids in milligrams per 100 grams of edible leaves of *L. cornuta* (Table 2). To the best of our knowledge, essential amino acids in *L. cornuta* leaves are reported in the current study for the first time. It is imperative to note that proteins are essential biological molecules with diverse functions, ranging from forming part of body structures to hormones, enzymes, signal transducers, and effectors, among others 37. Therefore, consuming appropriate amounts of essential amino acids through diet ensures proper functioning of the body processes and promotes health 38. Thus, the presence of various amino acids in the leaves of *L. cornuta* depicts this plant's federal and health-promoting benefits, which partly underpins its utilisation as a valuable nutritious vegetable.

**Table 2:** Composition of amino acids in *L. cornuta* leaves

Amino acid	Concentration (mg/100g)
Histidine	251.20 ± 2.00
Methionine	41.23 ± 2.70
Isoleucine	37.23 ± 0.90
Cysteine	37.17 ± 3.40
Tryptophan	35.20 ± 0.80
Phenylalanine	34.27 ± 0.70
Valine	33.63 ± 1.00
Threonine	32.57 ± 0.30
Leucine	32.57 ± 0.30
Tyrosine	30.23 ± 0.10

The values are presented as *x̅* ± *SEM* of triplicate analyses.

### Mineral element composition

The analysis of *L. cornuta* dry leaves revealed some essential minerals elements, as shown in Table 3. The concentration of sodium and magnesium are reported for the first time in the current study. However, other studies indicate a range of calcium concentrations of 0.107 – 2.14 mg/g, Iron 0.0113 – 0.262 mg/g, and Zinc 0.00056 – 0.00579 mg/g, which are significantly lower than those reported in this study 35,39-41. These differences may be attributed to differences in soil characteristics and composition from which the samples were obtained. Mineral elements are essential micronutrients required in the body in small quantities, where they play important roles in metabolism, proper growth, and general health 42. The deficiency of these micronutrients causes various disorders, some with deleterious sequelae 42,43. Thus, the presence of these micronutrients in the leaves of *L. cornuta* fosters their nutritional value, further supporting their utilisation as vegetables.

**Table 3:** Mineral element concentration in *L. cornuta* leaves

Mineral element	Concentration (µg /g dw)
Calcium (Ca)	820.493 ± 1.05
Sodium (Na)	464.154 ± 1.7
Magnesium (Mg)	430.140 ± 3.8
Iron (Fe)	285.080 ± 6.8
Zinc (Zn)	21.488 ± 0.06

The values are presented as *x̅* ± *SEM* of triplicate analyses.

### Quantitative phytochemical composition of L. cornuta leaf powder

The evaluation of *L. cornuta* leaves for phenols, flavonoids, alkaloids, and saponins revealed varied quantities (Table 4). The current study revealed, for the first time, the quantitative phytochemical percentage values of phenols, flavonoids, alkaloids, and saponins in *L. cornuta*. Kaigongi et al.^44^ reported the presence of phenols, flavonoids, alkaloids, and saponins by qualitative phytochemical screening. The alkaloids, a group of nitrogen-containing compounds that may consist of one or more nitrogen atoms, are important secondary metabolites. There is a broad spectrum of biological properties of alkaloids, including antiviral, antibacterial, anti-inflammatory, and anticancer 45.

**Table 4:** Quantitative phytochemical composition of *L. cornuta* leaves

Phytochemical	Concentration (%)
Phenols	13.07 ± 0.6
Flavonoids	11.59 ± 0.1
Alkaloids	3.27 ± 0.1
Saponins	2.19 ± 0.1

The values are presented as *x̅* ± *SEM* of triplicate analyses.

The alkaloids, a group of nitrogen-containing compounds that may consist of one or more nitrogen atoms, are important secondary metabolites. There is a broad spectrum of biological properties of alkaloids, including antiviral, antibacterial, anti-inflammatory, and anticancer 45.

The saponins reported in the current study are naturally occurring surface-active steroidal or triterpenoid glycosides with a distinctive foaming characteristic in aqueous preparations. Like other secondary metabolites, saponins benefit patients suffering from inflammatory conditions, immune disorders, cancer, type 2 diabetes, hepatic diseases, viral disease, hyperlipidemia, and cardiovascular diseases.

These bioactive amalgams possess pharmacologic activity against a broad spectrum of diseases 48, and the medicinal value of this plant may be attributable to them. Recently, the anti-inflammatory and analgesic efficacy of the aqueous root extract of *L. cornuta*, associated with flavonoids and phenols, among other phytochemicals, were reported^17^, thus valorising this plant as a potential source of armamentaria for various diseases.

Furthermore, we determined the concentration of phenols and flavonoids in the aqueous and methanolic leaf extracts of *L. cornuta*, based on earlier reports of their broad pharmacologic activities^17^,^49^-^51^, using a spectrophotometric method 30,31. As the resuits in Table 5 (interpolated from calibration curves: *Y* = 0.009210*x* + 0.09029: *R*^2^ = 0.9799 for total phenolic content and *Y* = 58.322*x* − 5.1975; *R*^2^= 0.9629 for total flavonoid content) indicate that the methanolic leaf extract of *L. cornuta* had significantly higher total phenolic content than the aqueous extract (P<0.05); however, no significant differences between the total flavonoid contents were observed between the two extracts (P>0.05). Phenols are an extensive and diversified set of secondary metabolites that include flavonoids, stilbenes, lignans, benzoic acid derivatives, and cinnamic acids, among others, which have in common at least one hydroxylated aromatic ring with antioxidant and anti-inflammatory responses of an organism and therefore help to protect against various oxidative stress-associated diseases, such as diabetes, inflammation, cardiovascular disorders, neurodegenerative disorders, and cancer 21, 22.

**Table 5:** Total phenolic and flavonoid content of *L. cornuta* leaf extracts

Extract	Total phenolic content (mgGAE/g of dry weight)	Total Flavonoid Content (mgCE/g of dry weight)
Aqueous leaf extract of *L. cornuta*	57.77±1.65^b^	8.00 ± 0.005^a^
Methanol leaf extract of *L. cornuta*	83.10±4.32^a^	7.99 ± 0.03^a^

The values are presented as *x̅* ± *SEM* of triplicate analyses. Values with the same lowercase letter within the same column are not significantly different (P>0.05), while those with different lowercase letters within the same column are significantly different (P<0.05) (Unpaired t-test statistic).

### In vitro antioxidant activity of the aqueous and methanolic leaf extract of L. cornuta

The aqueous and methanolic leaf extracts of *L. cornuta* revealed significant (P<0.05) efficacy in scavenging the 2,2-diphenyl-2-picryl-hydrazyl (DPPH) radical in a dose-dependent manner, as shown in Table 6. Further, the results showed that L-ascorbic acid has significantly higher DPPH radical scavenging activity than the plant extracts at all concentrations except at 0.1 µg/ml (P<0.05; Table 6). Besides, the methanolic leaf extract of L. cornuta had significantly higher DPPH radical scavenging activity than the aqueous extract at concentrations of 100 Ug/ml and 1000 µg/ml, respectively; however, at concentrations of 0.01 µg/ml through to 10 µg/ml, the aqueous leaf extract showed significantly higher DPPH radical scavenging activity than the methanolic extract (P<0.05; Table 6). Furthermore, we determined the concentrations of the aqueous and methanolic leaf extracts of *L. cornuta* and L-ascorbic acid, which could scavenge 50 % of the DPPH radical (IC_50_). The IC_50_ values were 681.57± 2.21 µg/ml, 72.96± 0.32 µg/ml, and 53.58± 0.21 µg/ml for the methanolic extract, aqueous extract and L-ascorbic acid, respectively (Table 6).

**Table 6:** DPPH radical scavenging activity of the aqueous and methanolic leaf extract of *L. cornuta*

Concentration (µg/ml)	% DPPH radical scavenging activity		

	Methanolic extract	Aqueous extract	L-Ascorbic acid
0.01	7.20 ± 0.29^E^_a_	1.21±0.09^E^_b_	7.44±0.30^C^_a_
0.1	17.66 ± 0.31^D^_a_	1.61±0.12^DE^_c_	9.68±0.40^C^_b_
1	20.96 ± 0.31^D^_b_	3.57±0.23^D^_c_	36.47±1.08^B^_a_
10	31.73 ± 2.44^C^_b_	14.96±0.94^C^_c_	43.69±3.37^B^_a_
100	46.55 ± 0.55^B^_c_	68.53±0.22^B^_b_	93.31±0.89^A^_a_
1000	73.36 ± 0.11^A^_c_	81.86±0.32^A^_b_	95.97±0.99^A^_a_

**IC**_**50**_ **(µg/ml)**	681.57± 2.21	72.96± 0.32	53.58± 0.21

The values are presented as *x̅* ± *SEM* of triplicate analyses. Means with dissimilar uppercase letters within the same column and those with dissimilar lowercase letters within the same row are significantly different (P<0.05; One-Way ANOVA with Tukey's post hoc test).

According to Minsas *et al.* 33, the antioxidant efficacies of the aqueous extract and L-ascorbic acid were considered strong. In contrast, the methanolic extract had very weak antioxidant efficacy, depicting the high extractive value of water in dissolving many antioxidant phytochemicals as per the earlier reports 17,33,54. Our results differ from those of Akimat *et al.* 17, who reported significantly higher antioxidant efficacy of the aqueous root extract of *L. cornuta*. These differences may be attributable to the differences in plant parts, the geographical location of the plant, and the season the samples were collected, which influence the type and concentration of phytocompounds 54.

Research shows that the antioxidant efficacy of plant extracts may be due to polyphenolic phytocompounds, such as flavonoids, phenols, tannins, and coumarins, among others, which either scavenge the free radicals, inhibit, or terminate the chain reactions for generating thisradicals^54^. Thus, based on our results, the antioxidant efficacy of the methanolic leaf extract may be through the modification of free radical-generating reaction cascades to avert their accumulation, while that of the aqueous extract may be via direct quenching of free radicals coupled with preventing their generation 54. The consumption of this herb as a vegetable may ultimately confer antioxidant efficacy to thwart oxidative stress in the body.

### Fourier transform infrared analysis of L. cornuta leaf powder

**Figure 2:**
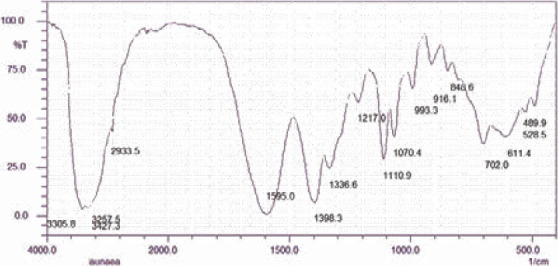
FTIR bands for functional moieties in *L. cornuta* leaf powder

The Fourier Transform Infrared spectra of *L. cornuta* leaf powder are displayed in Figure 2. The current study revealed the presence of 16 peaks of functional groups at 3427.3, 3305.8, 3257.5, 2933.5, 1595.0, 1398.3, 1336.6, 1217.0, 1110.9, 1070.4, 993.3, 916.1, 843.6, 702.0, 611.4, 528.5 and 489.9. The frequency ranges of FTIR absorbances were assigned to corresponding functional groups according to Wade 2006 55 and Wangia et al.^14^ as 3427.3 − 3257.5 (O-H for alcohol and phenols), 2933.5 (CH), 1595.0 (C=C for an aromatic ring), 1398.3 - 1336.6 (C-O for amide or C-C for phenyl groups), 1217.0 (C-O for carbonyl or OH bending or C-N nitriles), 1110.9-1070.4 (C-O for ester bond for sugars), 993.3, 916.1, and 843.6-702.0 (C-H for aromatic rings).

The recorded Fourier Transform Infrared spectra confirmed the composition of some of the phytochemicals of *L. cornuta* leaf powder. The band at 3427.3 cm^-1^ and 1595.0 cm-1 correspond to -OH and C=C groups, respectively, confirming the presence of phenolic compounds.^56^,^57^ The absorbance at 1217.0 cm^-1^ was assigned to the tertiary amine group, which frequently forms the alkaloid's nitrogen.^58^ The functional groups of saponins, −OH, C-H, C=C, and C-O-C, were revealed with characteristic FTIR absorbance bands at 3427.3, 2933.5, 1595.0, and 1217.0, respectively 34. The sapogenin glycosidic linkage was demonstrated by absorptions of C-O-C being indicative of monodesmosidic saponins ^59^.

## Conclusions and recommendations

Based on the findings from the current study, we conclude that *L. cornuta* leaves contain varying concentrations of nutritive and antinutritive constituents, with nutritional and health-promoting benefits, which partly validate its usage as food and folkloric utilisation in treating various diseases. In addition, the methanolic leaf extract of *L. cornuta* possesses strong antioxidant activity, which may explain the use of this plant in managing oxidative stress-associated like diabetes, cancer, chronic joint pain, and liver disease in herbal medicine. To the best of our knowledge, this is the first time the quantities of saponins, alkaloids, phenols, and flavonoids in the leaf powders and total phenolic and flavonoid content, together with the anti-oxidant activity of the leaf extracts, are reported. Our findings provide the initial background of science that explains the use of *L. cornuta* as food and medicine, and further studies to isolate and characterise bioactive compounds from this plant and determination of their modes of bioactivity should be undertaken.

## References

[1] Ülger G, Songur A N, Songur A N, Çırak O, Ülger T G (2018). Role of Vegetables in Human Nutrition and Disease Prevention Prevention Taha. Vegetables-Importance of Quality Vegetables to Human Health.

[2] Nachimuthu K, Jeyakumar P (2019). Medicinal properties of vegetable crops. Int J Chem Stud.

[3] Megaloudi F (2005). Wild and Cultivated Vegetables, Herbs and Spices Wild and Cultivated Vegetables, Herbs and Spices in Greek Antiquity (900 BC to 400 BC). Environmental Archaelogy.

[4] Abukutsa-Onyango M (2007). The diversity of cultivated African leafy vegetables in three communities in Western Kenya. African Journal of Food, Agriculture, Nutrition and Development.

[5] Misonge J O, Kinyanjui J G, Kingori W M, Mwalukumbi J M (2015). Phytochemical screening and cytotoxicity evaluation of Launaea Cornuta H. (Asteraceae) using brine shrimp. Merit Research Journal of Medicine and Medical Sciences.

[6] Grubben G J H, Denton O A (2004). Plant Resources of Tropical Africa 2 Vegetables.

[7] Onyango M A (2007). The diversity of cultivated African leafy vegetables in three communities in Western Kenya. African Journal of Food Agriculture Nutrition and Development.

[8] Kareru P G, Kenji G M, Gachanja A N, Keriko J M, Mungai G (2007). Traditional medicines among the Embu and Mbeere peoples of Kenya. African Journal of Traditional, Complementary and Alternative Medicines.

[9] Onyancha M J, Gikonyo K N, Wachira W S, Gicheru M M (2019). An ethnobotanical survey of plants used for the treatment and management of cancer in Embu County, Kenya. Journal of Medicinal Plants Studies.

[10] Mutembei J K (2018). Phytochemical and antimicrobial evaluation of selected medicinal plants in Meru community of Kenya. Journal of Medicinal Plants for Economic Development.

[11] Musila M F, Dossaji S F, Nguta J M, Lukhoba C W, Munyao J M (2013). In vivo antimalarial activity, toxicity and phytochemical screening of selected antimalarial plants. J Ethnopharmacol.

[12] Herdberg I (1982). Inventory of plants used in traditional medicine Tanzania. i. plants of the families acanthaceae- cucurbitaceae. J Ethnopharmacol.

[13] Boylan F, Menezes S, Leita G G (2015). Screening of Brazilian plant extracts for antioxidant activity by the use of DPPH free radical method. Phytotherapy Research.

[14] Kipkore W, Wanjohi B, Rono H, Kigen G (2014). A study of the medicinal plants used by the Marakwet Community in Kenya. J Ethnobiol Ethnomed.

[15] Kokwaro J O (2009). Medicinal plants of east Africa.

[16] Wambugu S N (2011). Medicinal plants used in the management of chronic joint pains in Machakos and Makueni counties, Kenya. J Ethnopharmacol.

[17] Akimat E K, Omwenga G I, Moriasi G A, Ngugi M P (2021). Antioxidant, Anti-Inflammatory, Acute Oral Toxicity, and Qualitative Phytochemistry of The Aqueous Root Extract of Launaea cornuta (Hochst. Ex Oliv. and Hiern.). J Evid Based Integr Med.

[18] Houghton P J, Raman A (1998). Laboratory Handbook for the Fractionation of Natural Extracts.

[19] Onyancha J M, Gikonyo N K, Wachira S W, Mwitari P G, Gicheru M M (2018). Anticancer activities and safety evaluation of selected Kenyan plant extracts against breast cancer cell lines. Journal of Pharmacognosy and Phytotherapy.

[20] Association of official Analytical Chemists (1990). AOAC: Official Methods of Analysis (Volume 1). Journal of the Association of Official Agricultural Chemist.

[21] Nielsen S S (2006). Proximate Assays in Food Analysis. Encyclopedia of Analytical Chemistry.

[22] Udofia N E, Onyancha J M, Mworia M, William N, Apiri M G (2020). Chemical Composition of Moringa oleifera Lam. and Moringa stenopetala Bac. Leaves from Kenya. International Journal of Plant Research.

[23] Okoronkwo N E, Mba K C, Nnorom I C (2017). Estimation of Protein Content and Amino Acid Compositions in Selected Plant Samples Using UV-Vis Spectrophotometeric Method. American Journal of Food Science and Health.

[24] Achi N K, Onyeabo C, Ekeleme-egedigwe C A, Onyeanula J C (2017). Phytochemical, Proximate Analysis, Vitamin and Mineral Composition of Aqueous Extract of Ficus capensis leaves in South Eastern Nigeria. J App/Pharm Sci.

[25] Harbone J B (1998). Phytochemical Methods: A guide to modern techniques of plant analysis.

[26] Ezeonu C S, Ejikeme C M (2016). Qualitative and Quantitative Determination of Phytochemical Contents of Indigenous Nigerian Softwoods. New Journa/ of Science.

[27] Devkota A, Sahu A (2018). Antimicrobial activities and phytochemical screening of leaf extract of Mikania micrantha HBK. J Nat Hist Mus.

[28] Kwada A D, Tella I O (2009). Determination of infochemicals and the phytochemical screening of the foliage and stembark of Senna siamea (lam.) in Yola, Adamawa State. Journal of Medicinal Plants Research.

[29] Hussain I (2011). Phytochemical analysis of selected medicinal plants. Afr J Biotechnol.

[30] Saeed N, Khan M R, Shabbir M (2012). Antioxidant activity, total phenolic and total flavonoid contents of whole plant extracts Torilis leptophylla L. BMC Complement Altern Med.

[31] Cosmulescu S, Trandafir I, Nour V (2017). Phenolic acids and flavonoids profiles of extracts from edible wild fruits and their antioxidant properties. Int J Food Prop.

[32] Minsas S (2020). Screening of Bioactive Compounds and Antioxidant Activity of Ale-ale Shellfish (Meretrix meretrix) Crude Extracts from West Kalimantan, Indonesia.

[33] Wangia C O (2018). Quantitative and Fourier Transform Infrared Analysis of Saponins from Three Kenyan Ruellia Species.

[34] Schippers R (2004). Launaea cornuta (Hochst. Ex Oliv. and Hiern) C. Jeffrey.

[35] Medicalnewstoday (2022). “Why Do We Need Dietary Fiber?”.

[36] Sarker M S K (2017). Moringa leaf meal as natural feed additives on the growth performance and meat quality of commercial broiler chicken. Asian Journal of Medical and Biological Research.

[37] Slavin J (2012). Dietary guidelines: Are we on the right path?. Nutr Today.

[38] Cordeiro L (2012). Household Dietary Diversity, Wild Edible Plants, and Diarrhea among Rural Households in Tanzania Household Dietary Diversity, Wild Edible Plants, and Diarrhea among Rural Households in Tanzania. Journal of Medicinally Active plants.

[39] Orech F O (2007). Mineral content of traditional leafy vegetables from western Kenya. Int J Food Sci Nutr.

[40] Weinberger K, Msuya J (2004). Indigenous Vegetables in Tanzania- Significance and Prospects.

[41] Raghavendra H, Kekuda T, Vijayananda B, Duressa D, Solomon T (2016). Nutritive Composition and Antimicrobial Activity of Moringa stenopetala (Baker f.) Cufod. J Adv Med Pharm Sa.

[42] Valdez-Solana M A (2015). Nutritional content and elemental and phytochemical analyses of moringa oleifera grown in Mexico. J Chem.

[43] Kaigongi M M, Dossaji S F, Musila F M (2014). Antimicrobial Activity, Toxicity and Phytochemical Screening of Four Medicinal Plants Traditionally Used in Msambweni District. J Biol Agric Healthc.

[44] Adamski Z, Blythe L L, Milella L (2020). Biological Activities of Alkaloids: From Toxicology to Pharmacology.

[45] Schoenlechner R, Siebenhandl S, Bergho E (2008). Pseudocereals. in Gluten-free cereal products and beverages 150-190.

[46] Mohan V R, Tresina P S, Daffodil E D (2015). Antinutritional Factors in Legume Seeds: Characteristics and Determination. Encyclopedia of Food and Health.

[47] Moriasi G, Nelson E, Twahirwa E (2021). In vitro Anti-Inflammatory, Antioxidant, and Qualitative Phytochemical Evaluation of the Phytexponent Preparation of Selected Plants Advanced Techniques in Biology and Medicine. Adv Tech Biol Med.

[48] Moriasi G, Ireri A, Ngugi M (2020). Cognitive-Enhancing, Ex Vivo Antilipid Peroxidation and Qualitative Phytochemical Evaluation of the Aqueous and Methanolic Stem Bark Extracts of Lonchocarpus eriocalyx (Harms.). Biochem Res Int.

[49] Moriasi G, Kibiti C, Ngugi M (2021). In Vivo Antidiabetic Efficacy, Mineral Element Composition, and Qualitative Phytochemistry of the Aqueous Leaf Extracts of Pentas zanzibarica (Klotzsch.) Vatke and Olea europaea subspecies africana (Mill.). Journal of Advanced Biotechnology and Experimental Therapeutics.

[50] Tangney C C, Rasmussen H E (2013). Polyphenols, inflammation, and cardiovascular disease. Curr Atheroscler Rep.

[51] Mileo A M, Nisticò P, Miccadei S (2019). Polyphenols: Immunomodulatory and therapeutic implication in colorectal cancer. Front Immunol.

[52] Moriasi G, Ireri A, Ngugi M P (2020). In vitro antioxidant activities of the aqueous and methanolic stem bark extracts of Piliostigma thonningii (Schum.). J Evid Based Integr Med.

[53] Wade LG (2006). Organic Chemistry.

[54] Nandiyanto A B D, Oktiani R, Ragadhita R (2019). How to read and interpret ftir spectroscope of organic material. Indonesian Journal of Science and Technology.

[55] Hasana H, Desalegn E (2017). Characterization and Quantification of Phenolic Compounds from Leaf of Agarista salicifolia. Herbal Medicine: Open Access.

[56] (2020). https://chem.libretexts.org.Bookshelves/Organic_Chemistry/Book%3A_Basic_Principles_of_Organic_Chemistry_(Roberts_and_Caserio)/23%3A_Organonitrogen_Compounds_I_Amines/23.02%3A_Naturally_Occurring_Amines.

[57] Almutairi M S, Ali M (2014). Direct detection of saponins in crude extracts of soapnuts by FTIR. Natural Product Research. Formerly Natural Product Letters.

